# Escaping a Nightmare: Successfully Retrieving a Fractured Guidewire During Percutaneous Coronary Intervention

**DOI:** 10.7759/cureus.62273

**Published:** 2024-06-12

**Authors:** Sanjay Kumar Sharma

**Affiliations:** 1 Department of Cardiology, Neo Hospital, Noida, IND

**Keywords:** neurovascular remodeling device, solitaire, retrieval, percutaneous coronary intervention, guidewire fracture

## Abstract

A guidewire fracture seldom occurs as a complication of percutaneous coronary intervention (PCI). Guidewire fragments retained in the coronary tree can result in thrombosis, embolic phenomena, dissection, perforation, and vessel occlusion. This study represents a rare incidence of fractured guide wire, which occurred during PCI in a 44-year-old male due to the acute angle and heavy calcification which was safely and successfully retrieved using a 4×40 mm Solitaire device (Irvine, CA: Medtronic) (neurovascular remodeling device).

## Introduction

Angioplasty guidewire fracture is a very rare complication of percutaneous coronary intervention (PCI). The incidence of such complications in PCI is reported to range from 0.1% to 0.8% [1]. The approach to fractured guidewire management encompasses a range from conservative management to interventional and surgical strategies, depending on the location, chronicity, and clinical situation of the patient [2]. Removing such fractured guidewires from the lumen of coronary arteries presents significant challenges. Inability to do so may lead to devastating consequences including restenosis, embolism, perforation, new myocardial infarction, and ultimately, death. To remove such broken wire securely, proper technique and expertise are very much essential [3]. In the present case, an incidence of a broken guide wire during percutaneous transluminal coronary angioplasty (PTCA) procedure has been reported which was successfully and safely removed using a Solitaire device (Irvine, CA: Medtronic).

## Case presentation

**Figure 1:**
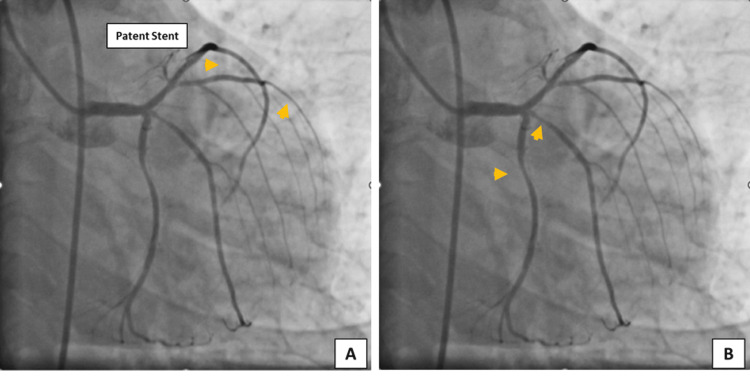
Angiogram showing (A) 80% stenosis in left circumflex artery and (B) 90% stenosis in ramus intermedius.

A 44-year-old male with a history of recent acute coronary syndrome (anterior wall myocardial infarction) and primary PTCA to left anterior descending artery (LAD) was presented with sudden onset of severe chest pain and breathlessness at our tertiary care center. On admission, the patient’s body temperature was 98.6°F, pulse was 78/minute, blood pressure was 90/60 mmHg, and oxygen saturation (SPO_2_) was 97%. Electrocardiogram showed left ventricular hypertrophy, biphasic T-wave in V1-V2, and T-wave depression in V4-V6 leads. A coronary angiogram revealed a patent stent in LAD, severe stenosis (80%) in left circumflex artery (LCX), 90% stenosis in ramus intermedius, and a normal right coronary artery (Figures 1A, 1B). On strong suspicion of in-stent thrombosis, the patient was immediately shifted to the catheterization laboratory, late at night. The troponin value at the time of admission was 1.4 ng/mL. The patient was diagnosed with non-ST-elevation myocardial infarction with cardiogenic shock and severe left ventricular dysfunction with a 30% ejection fraction.

**Figure 2:**
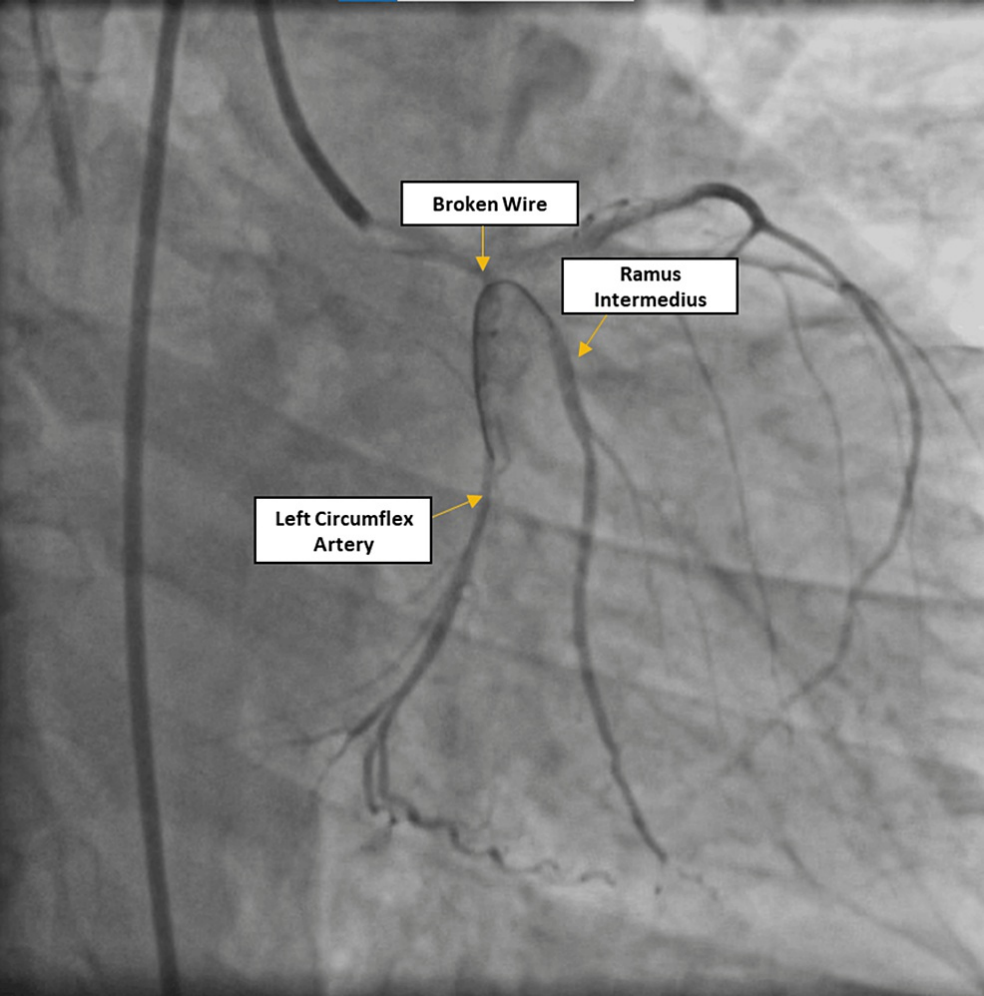
Broken wire in “U” shape between ramus intermedius and left circumflex artery.

**Figure 3:**
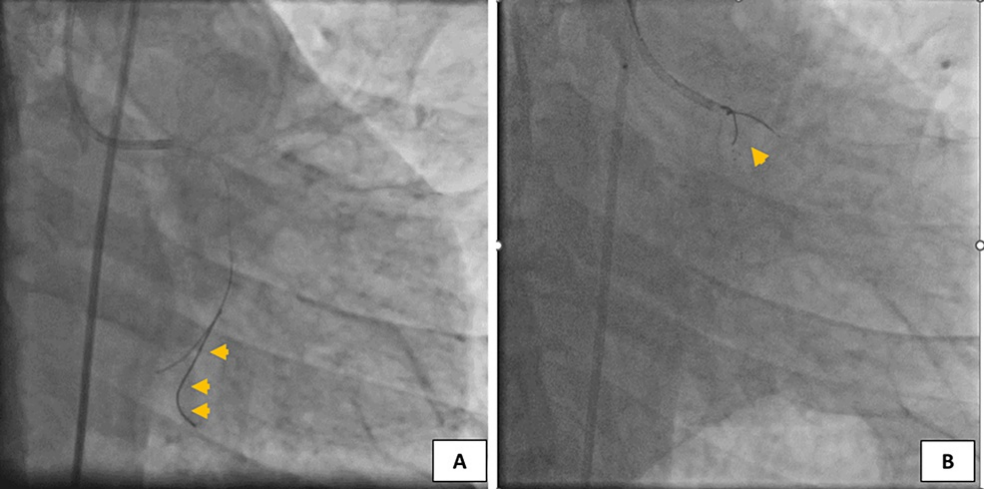
Balanced Middle Weight guidewire spontaneously broke in the left circumflex artery and embolized distally (A). Using a 4×40 mm Solitaire device (Irvine, CA: Medtronic), the broken guidewire was successfully removed (B).

PTCA was planned for LCX and ramus intermedius lesions. The PTCA procedure was carried out through femoral access and the left coronary artery was engaged with 6 Fr extra-backup (EBU) 3.5 guiding catheter, while a 0.014” Balanced Middle Weight (BMW) PTCA guidewire (New Delhi, India: Abbott Vascular) was used to cross the LCX. However, the anatomy of the LCX was unfavorable as it was arising at a very acute angle from the left main coronary artery. Due to the acute angle and heavy calcification, the BMW guidewire spontaneously broke in the LCX during PTCA and was embolized distally (Figure 2). An immediate decision was made to remove the broken guidewire; however, all the attempts to retrieve the broken guidewire using conventional hardware failed. Therefore, the operator uses a quick and bold hack of making use of already existing things, i.e., use of a 4×40 mm Solitaire revascularization device (Irvine, CA: Medtronic), generally used as a neurovascular remodeling device, to retrieve the broken guidewire from the coronary artery (Figure 3 and Video 1). Gratefully, the trick worked, and the broken guidewire was retrieved successfully without any further complications.

**Video 1:** Broken guidewire retrieved using a 4×40 mm Solitaire device (Irvine, CA: Medtronic).

**Figure 4:**
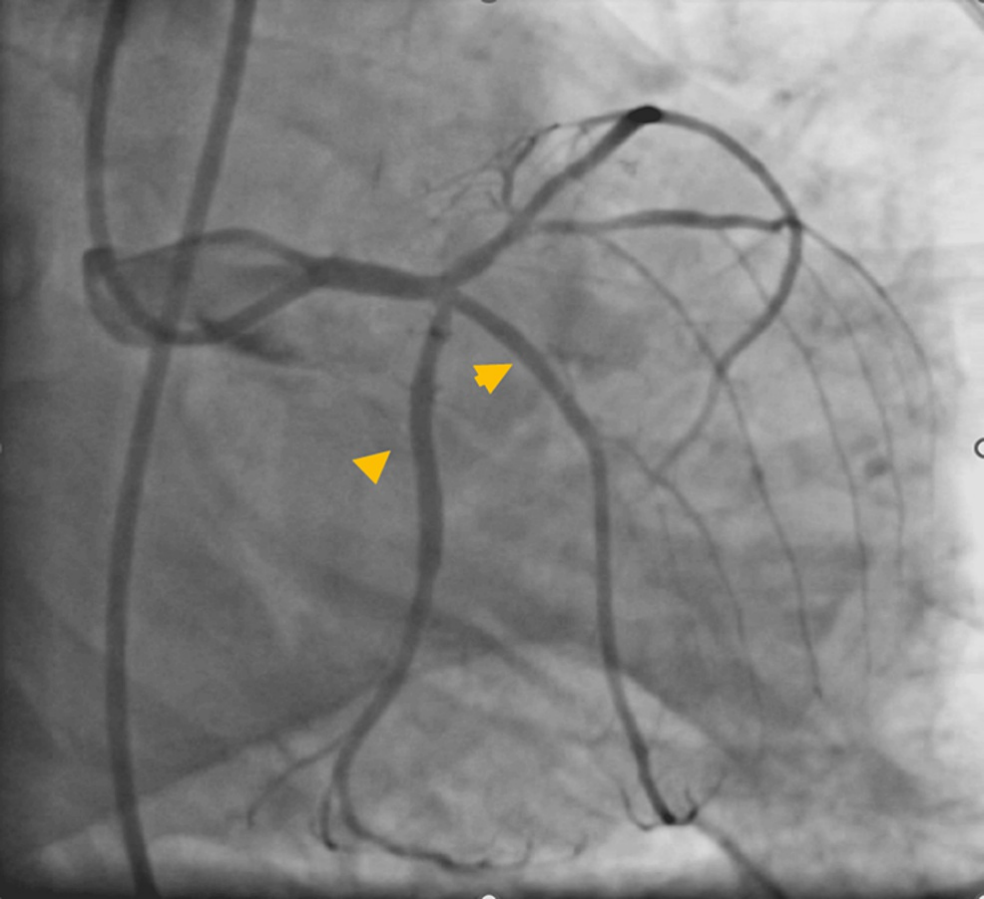
Stenting in left circumflex artery and ramus intermedius.

Later, the LCX was predilated using a 2.0×10 mm balloon at 14 atm, and a 2.75×20 mm Tetriflex sirolimus-eluting stent (Surat, India: Sahajanand Medical Technologies Ltd.) was deployed at 12-14 atm in LCX. Post dilatation was done using a 3.0×10 mm balloon at 12-14 atm and the blood flow was restored. Another Tetriflex sirolimus-eluting stent of 2.5×12 mm was deployed in the ramus intermedius at 11-12 atm and the blood flow was restored (Figure 4). Post procedure, the patient was shifted to the intensive coronary care unit for further observation and management. The patient recovered without any adverse events and was discharged after 72 hours of the angioplasty procedure with the recommendation of one-year dual antiplatelet therapy. Post discharge, the patient was asymptomatic at 15 days and one-month follow-up.

## Discussion

During PCI, fragments of the interventional guiding equipment may break and stay inside the coronary system which can increase the risk of thrombosis, embolic manifestations, ischemia, perforation, dissection, and vessel occlusion if remain inside the coronary arteries [4]. Factors contributing to the occurrence of a fractured guidewire during PCI include vigorous traction on the guidewires, arterial spasm, presence of fibrous calcified plaque, inadvertent handling of the catheter, and manufacturing defects. In addition, the use of bulky devices such as aspiration catheter, rotablator, and AngioJet catheter (Marlborough, MA: Boston Scientific Corp.) can increase the chances of this complication [3].

The basic composition of a guidewire includes a central core or a shaft, a spring coil, and coating. The typical location where the guidewire tends to break is the junction between the flexible 3 cm tip and the remainder of the guidewire [5]. Guidewires are entrapped frequently behind stent struts within the side branches. It occurs when the side branch originates at 90° angle or when there is a significant plaque shift [5]. The primary therapeutic option for the incidence of broken guidewire during PCI is interventional, retrieved by means of specialized harvesting devices such as filter wires, additional guidewire manipulation, snares, and retrieval forceps [2]. Hence, it is essential for the interventionists to be acquainted with various techniques for retrieving fractured and dislodged devices.

Management options include percutaneous removal using snare, or bioptome, entrapping the unraveled wire in the guide catheter with balloon inflation, and retrieving it with a twisting wire technique. Furthermore, fractured wire can be looped into the snare and thereby can be safely retrieved [6]. One can take two or more guidewires to intertwine the fractured guidewire and then try to bring it back into the guide catheter. One option is to attempt passing a second guidewire and then dilating it with a small semi-compliant balloon to disentangle it from stent struts. If the interventional retrieval fails and the signs of ischemia persist, the patient should be promptly referred for surgical intervention [5].

The optimal approach depends on the clinical scenario, size and nature of the fragment, anatomical considerations, severity of coronary artery disease, and the position where it is stuck. In the present case, recognizing the need for immediate intervention, the interventional team opted to retrieve the broken guidewire using an innovative approach. A 4×40 mm Solitaire device, which is typically used for thrombectomy in acute ischemic stroke cases, was considered due to its retrievable loop design and potential for capturing foreign objects within the vessel [7].

Although the primary indication for the Solitaire device is in neurovascular interventions for stroke, its adaptability and effectiveness have led to the exploration of its potential use in other medical scenarios, such as the retrieval of foreign objects like broken guidewires from coronary arteries during PCI [7-9]. However, such off-label use requires careful consideration and evaluation due to differences in vascular anatomy and characteristics between neurovascular and cardiovascular scenarios.

The prompt coordination of a skilled interventional team, utilization of specialized retrieval equipment, and meticulous technique played pivotal role in the successful removal of the broken guidewire. The successful retrieval of a broken guidewire using a Solitaire device highlights the adaptability of medical tools across different medical disciplines.

## Conclusions

In conclusion, the management of fractured guidewires during PCI poses significant challenges and requires a tailored approach based on various factors including clinical presentation, anatomy, and characteristics of the fragment. This study presented a successful retrieval of a broken guidewire during PCI using an innovative approach involving a Solitaire device, originally designed for thrombectomy in acute ischemic stroke cases. The adaptability and effectiveness of the Solitaire device in capturing foreign objects within the vessel demonstrate its potential utility in diverse medical scenarios, albeit with careful consideration and evaluation due to differences in vascular anatomy and characteristics.

The prompt coordination of a skilled interventional team, along with the utilization of specialized retrieval equipment and meticulous technique, played a crucial role in the favorable outcome of this case. This successful intervention underscores the importance of innovative approaches and interdisciplinary collaboration in overcoming challenging situations during PCI procedures.
